# Testing adequacy for DNA substitution models

**DOI:** 10.1186/s12859-019-2905-3

**Published:** 2019-06-20

**Authors:** Wei Chen, Toby Kenney, Joseph Bielawski, Hong Gu

**Affiliations:** 1 Department of Mathematics and Statistics, Dalhousie University, Halifax, Canada; 2 Department of Biology, Dalhousie University, Halifax, Canada

**Keywords:** Model adequacy test, DNA substitution model, Pearson’s goodness-of-fit test, Long branch attraction

## Abstract

**Background:**

Testing model adequacy is important before a DNA substitution model is chosen for phylogenetic inference. Using a mis-specified model can negatively impact phylogenetic inference, for example, the maximum likelihood method can be inconsistent when the DNA sequences are generated under a tree topology which is in the Felsentein Zone and analyzed with a mis-specified or inadequate model. However, model adequacy testing in phylogenetics is underdeveloped.

**Results:**

Here we develop a simple, general, powerful and robust model test based on Pearson’s goodness-of-fit test and binning of site patterns. We demonstrate through simulation that this test is robust in its high power to reject the inadequate models for a large range of different ways of binning site patterns while the Type I error is controlled well. In the real data analysis we discovered many cases where models chosen by another method can be rejected by this new test, in particular, our proposed test rejects the most complex DNA model (GTR+I+ *Γ*) while the Goldman-Cox test fails to reject the commonly used simple models.

**Conclusions:**

Model adequacy testing and bootstrap should be used together to assess reliability of conclusions after model selection and model fitting have already been applied to choose the model and fit it. The new goodness-of-fit test proposed in this paper is a simple and powerful model adequacy testing method serving such a regular model checking purpose. We caution against deriving strong conclusions from analyses based on inadequate models. At a minimum, those results derived from inadequate models can now be readly flagged using the new test, and reported as such.

## Background

The performance of phylogenetic methods relies on how well the model assumptions are satisfied. In reality, models are all mis-specified since the unknown underlying processes that generate the data are inevitably very complicated. What we could hope is that inference is still valid if the model mis-specification is not severe. For estimation of tree topologies, mis-specified models could lead to inconsistent estimates or inaccurate estimates of its bootstrap support [4, 12, 14, 39]. The inconsistent estimation of tree topologies typically happens when the model is oversimplified and the underlying true tree has the so-called “long branch attraction” problem where the unknown true tree contains long branches separated by a short internal branch [1, 13, 20, 21, 33, 34]. Estimation of branch lengths could also be heavily influenced by the selected substitution models, which in turn affects any downstream analyses that rely on the branch length estimates, such as divergence time estimates [32].

In order to be complete, a statistical analysis should not only provide estimates for the unknown parameters, but should also offer an assessment of the reliability of these estimates. There are two aspects of this assessment. Firstly, there is uncertainty due to limited data size i.e. variability in our estimates. This is assessed by a range of statistical tools. For biological applications the bootstrap is often used for this purpose. The second aspect of reliability assessment is model adequacy. If the model does not fit the data, then its results will be unreliable and may possibly lead to false biological conclusions. The techniques for assessing this are model adequacy tests, and have been greatly underdeveloped in phylogeny. Of course, certain model mis-specifications may lead to very small bias in certain estimates, but without knowing the true model, we do not know whether the mis-specification will lead to a bias in the estimates of interest. Some research into posterior predictive simulations [3, 7] has speculated that tests could be developed to be particularly sensitive to model mis-specifications that cause misestimation of topology. However, there is as yet insufficient evidence that such a technique performs better than general model adequacy testing. These two aspects of reliability assessment are both needed as they provide different insights into how the estimates may be unreliable. The bootstrap assesses whether there is enough data to support the conclusions reached. The model adequacy test assesses whether the model could plausibly have generated the data. In the case of weak bootstrap support, longer sequences are needed. (At least in theory, if longer sequences are not available, it simply means we do not have sufficient data to be sure of the true tree). In the case of an inadequate model, better models are needed. Analyzing a large data set with a wrong model could result in strong bootstrap support to biased conclusions.

In addition to the improved understanding of the reliability of our estimates, model adequacy testing can also provide valuable insights into the underlying biological processes. For example, if a model without rate variation among sites is adequate, for a reasonably large dataset, it suggests that the among-site variation is likely to be small for this sequence. Examples of work on inferring aspects of the biological processes from model adequacy include [9, 10, 19].

Model adequacy tests are an area that has been greatly underdeveloped in phylogeny with a relatively small number of related publications in the literature (e.g. [2, 3, 8, 17, 30, 38]) and it is not at all a common practice for researchers to validate their models by checking the model adequacy. There are several reasons why adequacy testing is underdeveloped for molecular phylogenetics. Firstly, the substitution models for molecular data are very different from the typical models in the statistical literature, so off-the-shelf methods cannot be immediately applied in the way that other methods (e.g. bootstrapping) can be applied to phylogenetics. Second, there appears to be widespread misunderstanding of the purpose of model adequacy testing. Many biologists incorrectly think of model adequacy testing as an alternative to model selection. This confusion may come partly from the tendency in the literature to categorise model assessment into (1) relative model fit and (2) absolute goodness of fit. While these both have the ability to reject some models, relative model fit inherently suggests a better model, and thus naturally leads to model selection methods. It is however limited by the choice of alternative models, and the constraint that it should choose exactly one model. Without a suitable choice of alternative model, model selection is left choosing the least bad model from the candidates, with no warning that the model should not be used. Parameter estimates under the selected model could be highly biased, leading to the wrong biological conclusions. Model adequacy testing, on the other hand, provides an objective measure of whether the selected model is suitable for analyzing the data, even though the model is an idealized version of the true process. Thus, model adequacy testing provides an additional level of support for conclusions that cannot be obtained from the mere process of model selection.

Powerful adequacy tests for DNA substitution models are almost nonexistant in the phylogenetic literature [16, 22]. The most well known frequentist model adequacy test is the Goldman-Cox (GC) test [17] which uses the likelihood ratio test statistic between the multinomial distribution and the model in question as a test statistic. An alternative method is Bayesian posterior predictive simulations (PPSs) [2]. Unfortunately, using both simulated and real-data, both of these tests were demonstrated by [30], as lacking power to reject models simpler than the optimal models selected by any standard model selection criterion (hLRT, AICc, BIC and DT) [27, 28]. The results in [30] also suggest that the GC test is generally slightly more powerful than the PPS test, but Waddell et al. [38] showed that the GC test generally lacks power. Interestingly, the two possible exceptions to the problem of low power were obtained by Waddell et al. [38] through marginalization of the site patterns. The first marginal test assessed the reversibility assumption through symmetry of the pairwise frequency matrices of each pair of taxa. The second marginal test employed the idea of binning of site patterns to ensure the appropriateness of the Chi-squared test. However, the power of these two marginal tests was demonstrated only in a single example.

The purpose of this study is to address the problem of power when testing the adequacy of DNA substitution models. We propose a simple, powerful and robust model adequacy test based on Pearson’s goodness-of-fit test (*X*^2^). Our method is also based on binning of site patterns, but our method avoids aspects of the Waddell et al. [31] method that might explain why it has undergone no further development. Specifically, Waddell et al. [31] (i) performed binning based on the parsimony scores of the site patterns relative to the maximum likelihood (ML) tree (under the null model in the test) and (ii) employed seemingly arbitrary methods to ensure the well-known rule-of-thumb of the *X*^2^ test (that the expected number of samples in each bin is at least 5) was satisfied. Our method employs a K-means clustering method for binning that leads to a generally powerful test for DNA substitution model adequacy. The test is general, rather than focused on any single aspect of model mis-specification. We use both simulation and real data analysis to evaluate the new method, and we discuss the joint use of bootstrapping and model adequacy tests as a general means to improve phylogenetic inference.

## Methods

### A review of the GC test

The Goldman-Cox test (GC test) [17] for testing the adequacy of a substitution model is based on the likelihood ratio test (LRT) statistic between the multinomial distribution and the model in question. In principle, the likelihood ratio statistic has an approximate *χ*^2^ distribution with degrees of freedom equal to the number of patterns minus the number of estimated parameters in the model. However, two reasons prevent the use of a *χ*^2^ distribution. The first is the *χ*^2^ approximation requires that each attainable site pattern should appear in the sample a few times. This requirement is usually not satisfied for the real sequence data, since the number of possible patterns is very large (4^*n*^ with *n* being the number of taxa for DNA data) and a large proportion of the site patterns are constant for the alignments used in phylogenetic analysis. Thus many patterns are not observed in the data and many observed informative patterns have very low frequencies. The second reason that complicates the degree-of-freedom issue is that it is hard to determine what degree-of-freedom should be counted for the phylogenetic tree estimated in the null hypothesis. To assess the null hypothesis, the GC test employs a parametric bootstrap to simulate a set of sequences based on the maximum likelihood tree with the maximum likelihood estimates (MLE) for all parameters in the null model from the original data. The test statistic is calculated then for each simulated data set and they form the null distribution. Note that for this method the maximum likelihood tree and all the parameters under the substitution model of the null hypothesis need to be estimated for each simulated data set, which makes this test computationally expensive, especially for large numbers of taxa. It could also be less accurate when the search for the maximum likelihood tree topology does not return the global maximum of the likelihood. Unfortunately, despite the amount of compuation involved, the test was shown to be lack of power in rejecting the tested models [30, 38].

The same two reasons that preclude the use of a *χ*^2^ distribution for the LRT statistics also preclude the use of the Pearsons goodness-of-fit test (*X*^2^) for such problems. Binning of the site patterns can typically remedy the problem of low site-pattern counts for using a Pearsons goodness-of-fit test.

### Pearsons goodness-of-fit test through binning of site patterns

As a goodness-of-fit test, the Pearson’s *χ*^2^ test compares the observed frequency distribution and the expected frequency distribution under the null hypothesis for categorical data. The null hypothesis for testing a DNA substitution model is:

*H*_0_: The substitution model $\mathcal {M}$ is the true model.

Tree topology is a nuisance parameter here. The test statistic is:

$$X^{2}=\sum_{i=1}^{K}\frac{(O_{i}-E_{i})^{2}}{E_{i}} $$

where *O*_*i*_ and *E*_*i*_ are the observed frequency and the expected frequency of the *i*th category and *K* is the number of categories. The test statistic is compared against the *χ*^2^ distribution with *K*−1 degrees of freedom.

In principle, the test is applicable for any procedure that bins the sites such that the standard rule-of-thumb for this test is satisfied. However the power of the test differs for the different binning procedures. If model $\mathcal {M}$ is wrong, there will be some elements in the estimated DNA substitution matrix that are biased, which in turn will cause the estimated probabilities of some site patterns to be too high and some other site pattern probabilities to be too low. Our procedure bins the site patterns such that most bins are comprised mostly of sites with estimated probabilities biased up or mostly of sites with estimated probabilities biased down.

The rule-of-thumb of Pearson’s *χ*^2^ test requires that no more than 20% of the bins have expected frequencies below 5. In fact, it is not hard to meet these requirements if we bin the site patterns into a reasonable number of bins. Generally speaking, this test is applicable for any arbitrarily chosen rules for binning the site patterns. The question is how to bin the site patterns such that the power of the test can be optimized.

Given that tree topology is a nuisance parameter, a statistic that is most directly related to the site-pattern probabilities is the observed frequencies of the nucleotide characters, $\hat \pi = (\hat \pi _{A}, \hat \pi _{C}, \hat \pi _{G}, \hat \pi _{T})$, at a site. This will provide a basic binning step directly, i.e. the sites with the same frequency summary statistics are first binned together. For example for the 4-taxon case, sites (A, C, C, A), (C, A, C, A), (A, C, A, C) etc. will have the same summary statistics, and will be binned together. For the parsimoniously uninformative sites, this agrees with the basic binning used by [38]. We will illustrate this equal frequency binning method based on 4-taxon trees. For cases with a large number of taxa, we further bin sites with similar frequency vectors based on a clustering method. In this paper, we use the simple *K*-means clustering method.

### Equal frequency binning for a 4-taxon tree

For a 4-taxon tree, there are 256 different site patterns possible. These site patterns can be summarized by the following five different types according to the proportions of nucleotide characters (see Table 1). For example, sites XXYY and XYYX (where X and Y are any two distinct nucleotides) are the same type, because the proportions of X and Y are both $\frac {1}{2}$, and sites XXXY, XXYX are the same type because the proportions of X and Y are $\frac {3}{4}$ and $\frac {1}{4}$ respectively. Each type of site pattern contains a different number of bins depending on the nucleotide characters occupying X, Y, Z, and W.

**Table 1:** Different types of site patterns and their corresponing numbers of bins for a 4-taxon tree

Type A:	XXXX;	4 bins
Type B:	XXYY, XYYX, XYXY;	6 bins
Type C:	XXXY, XYXX, XXYX,	
	YXXX;	12 bins
Type D:	XXYZ, XYZX, YZXX,	
	XYXZ, ZXYX, ZXXY;	12 bins
Type E:	XYZW.	1 bin

There are 35 bins in total. With this binning, the goodness-of-fit test procedure is:

1. Calculate *O*_*i*_ as the observed count of the *i*th bin.
2. Compute the ML tree and MLE of parameters under the null model.
3. Calculate the expected probabilities of site patterns based on the ML tree and MLE of model parameters.
4. Calculate expected probabilities, *P*_*i*_, for each bin and calculate the expected frequency *E*_*i*_=*nP*_*i*_, where *n* is the sequence length.
5. The test statistic:
  $$X^{2} = \sum_{i=1}^{35}\frac{(O_{i}-{nP}_{i})^{2}}{{nP}_{i}}, $$
  is compared to a *χ*^2^ distribution with df=34.

We employ simulation to illustrate the effects of equal frequency binning and the rationale of this test procedure in the “Results” section.

### The general frequency based binning model test

When *m* is the number of taxa, there are 4^*m*^ different site patterns, and ${m+3}\choose 3$ different frequency vectors. Binning based on exact equal frequency vectors is not practical for large *m* values. Also when the sequence length is small, even for 4-taxon case, the rule-of-thumb of Pearson’s *χ*^2^ test may not be satisfied when using the exact equal frequency binning procedure. The idea is then extended so that sites with similar frequency vectors will be binned together. The *K*-means clustering method is used for this purpose.

In data mining, *K*-means clustering is a simple approach for clustering the observed (vector valued) data into different clusters according to their similarity, often measured by the Euclidian distance. Since site patterns are summarized by numerical values, it is easy to cluster these frequency vectors using any standard clustering method.

In this case, computing the expected frequencies exactly would involve summing over all possible site patterns. This is clearly not feasible for larger numbers of taxa. We therefore estimate the expected frequencies empirically. By simulating a very large number of sites, and assigning each site to the nearest bin, we can quickly obtain a good estimate for the probability of each bin under the null model. We will discuss what a “very large number” should be in the following subsection and conclude that for most practical purposes, we should simulate between 100,000 and 1,000,000 sites.

#### The Goodness-of-fit test Procedure in general

1. Summarize each site pattern into a frequency vector *f*_*i*_=(*f*_*Ai*_,*f*_*Ci*_,*f*_*Gi*_,*f*_*Ti*_),*i*=1,2,⋯,*n* and create an *n*×4 matrix:
  $$F = \left(\begin{array}{cccc} f_{A1} & f_{C1} & f_{G1} & f_{T1} \\ f_{A2} & f_{C2} & f_{G2} & f_{T2} \\ \vdots & \vdots & \vdots & \vdots \\ f_{An} & f_{Cn} & f_{Gn} & f_{Tn} \\ \end{array}\right) $$
  where each row contains the frequencies of observed nucleotides for the corresponding site.
2. Use the *K*-means clustering approach for binning the rows in matrix *F* into *K* clusters.
3. For *j*=1,2,⋯,*K*, denote the center of the *j*th bin by *C*_*j*_. Calculate the observed frequency for the *j*th bin, *O*_*j*_, as the total number of sites assigned to the *j*th bin.
4. Compute the ML tree and the MLE for all parameters under the Null model.
5. Use a parametric bootstrap to simulate an extremely long (*M* sites) DNA sequence data *X*^∗^ based on the ML tree and the MLE of model parameters.
6. From sequence data *X*^∗^, calculate the *M*×4 frequency matrix *F*^∗^, where each row contains the frequencies of nucleotide characters of each site:
  $$F^{*} = \left(\begin{array}{cccc} f_{A1}^{*} & f_{C1}^{*} & f_{G1}^{*} & f_{T1}^{*} \\ f_{A2}^{*} & f_{C2}^{*} & f_{G2}^{*} & f_{T2}^{*} \\ \vdots & \vdots & \vdots & \vdots \\ f_{AM}^{*} & f_{CM}^{*} & f_{GM}^{*} & f_{TM}^{*} \\ \end{array}\right) $$
7. Cluster the rows in *F*^∗^ to the original *K* clusters found in step 2 by comparing the Euclidian distance of each row to the *K* centers (*C*_1_,*C*_2_,⋯,*C*_*K*_) calculated in step 3 and assign the row to the cluster with the smallest Euclidian distance. Denote the number of rows assigned to the *j*th bin by *S*_*j*_. Then, the expected size of the *j*th bin, *E*_*j*_, can be calculated as:
  $$E_{j}=\frac{{nS}_{j}}{M}$$
  where *n* is the sequence length in the observed data set.
8. The test statistic is:
  $$X^{2} = \sum_{j=1}^{K} \frac{(O_{j}-E_{j})^{2}}{E_{j}}$$
  Under *H*_0_, *X*^2^ follows the *χ*^2^ distribution with df=*K*−1

### How many sites do we need to simulate to estimate the probability?

Because we do not calculate the true probabilities exactly, our simulation will introduce some error in our calculated chi-squared statistic. We are able to bound this error for large sample sizes.

#### **Theorem 1**

If the data include *n* sites, we divide the site patterns into *K* bins which satisfy the rule of thumb that the expected number of sites in each bin is at least 5, and we simulate *M* points, then the mean squared error in our estimated chi-squared statistic due to this simulation is at most

$$\frac{16Kn}{25M}$$

The proof of this theorem is in Appendix B. Recall that our *p*-value comes from a chi-square distribution with *K*−1 degrees of freedom. If the error in our statistic is *E*, then the error in our *p*-value is approximately ${Ef}_{\chi ^{2}_{K-1}}(X^{2})$, that is, the error in our chi-squared statistic multiplied by the density of the chi-squared distribution at the observed statistic value. Since we are interested in getting accurate *p*-values near the critical value, we can calculate the error in the *p*-values under the assumption that *X*^2^ is near the critical value. For reasonably large *K*, we have that the chi-squared distribution is approximately normal with variance 2(*K*−1), which means that the density at the critical value is approximately $\frac {e^{-\frac {\Phi ^{-1}(\alpha)^{2}}{2}}}{2\sqrt {\pi (K-1)}}$. For *α*=0.05, this is approximately $\frac {0.0292}{\sqrt {K-1}}$. We can then choose *M* to control the mean squared error near the critical value. For example to ensure that the root mean squared error in the *p*-value is at most 0.005 for *p*-values near 0.05, we would solve

$$\begin{array}{*{20}l} \frac{0.0292^{2}}{K-1}\times\frac{16Kn}{25M}&=0.005^{2}\\ M&=22\frac{Kn}{K-1} \end{array} $$

For our examples with *n* at most a few thousand, this means that *M*=100,000 should give sufficiently accurate *p*-values near the critical value.

### Choosing the number of bins

Another question not fully addressed is how to choose the number of bins *K*. For the classical *K*-means method, there are a number of standard approaches for choosing *K*. However, the purposes behind those methods are very different from our purposes, so those methods may not be applicable to our test. The reason we use a binning procedure in the first place is that using all possible site patterns leads to violation of the rule-of-thumb for applying Pearson’s Chi-squared test (and also the infeasably large number of site patterns for large numbers of taxa causes computational issues). If this problem did not arise, then taking all possible site patterns would be the natural thing to do. This therefore suggests that taking *K* as large as possible while retaining these rule-of-thumb would be the best thing to do. The trouble with this is that the standard rule-of-thumb does not correspond to precise boundaries, so the largest value of *K* that does not violate the rule-of-thumb varies according to the data and the model being tested. Further, as *K* gets larger, the accuracy of the Chi-squared approximation decreases. It therefore makes sense to consider the *p*-values for a range of suitably large *K*. Our simulation studies and real data analyses show that in most cases, sufficiently large values of *K* give similar conclusions, so the exact choice of *K* does not matter too much.

### Degrees of freedom

The asymptotic Chi-squared distribution of Pearson’s Chi-squared statistic is based on a number of approximations, which may not be totally appropriate in our case. A lot of work has been done on the appropriate asymptotics in these cases [25]. For simplicity, we have used the standard Pearson statistic, and used *K*−1 degrees of freedom. This is conservative, so should lead to a smaller rate of Type I errors. Given the power we achieved with this method (see Results), it seems that this will be sufficient for most cases. However, there are potentially several possibilities to increase the power for this test which will be discussed below.

The use of *K*−1 degrees of freedom ignores that the parameter values are estimated from the data. If the parameter values were estimated from just the frequency of each bin, then we would have a Chi-squared distribution with *K*−*d*−1 degrees of freedom, where *d* is the number of parameters estimated. However, the parameter values are estimated using the site patterns, rather than just the bins, which for many models leads to different parameter estimates. The theory behind this case was studied by Chernoff and Lehmann [5], and the asymptotic distribution of the Chi-square statistic is the distribution of $\chi ^{2}_{(K-d-1)}+\sum _{i=1}^{d} \lambda _{i}Z_{i}^{2}$, where the $Z_{i}^{\prime }s$ are independent standard normal variables and $\lambda _{i}^{\prime }s$ are the eigenvalues of the matrix (*I*−*BJ*^−1^*B*^*T*^). The matrix *J* is the information matrix of the parameter estimates, and *B* is the *K* × *d* matrix with *i*,*k*th entry $\frac {1}{\sqrt {p_{i}}}\frac {\partial p_{i}}{\partial \theta _{k}}$, where *p*_*i*_ is the probability of the *i*th bin and *θ*_*k*_ is the *k*th parameter in the model. If the parameters were estimated according to the bins rather than the site patterns, we would have *λ*_*i*_=0 for all *i*, and the asymptotic distribution would be Chi-squared with *K*−*d*−1 degrees of freedom. The values of the *λ*_*i*_ depend on the true parameter values, so we do not get an asymptotic distribution which works in general. It also involves computing the relevant matrices.

Another way to remedy this problem and get a more accurate distribution of the test statistic, due to Rao and Robson [29], is to use a different quadratic form of the statistic, *X*^2^=*V*^*T*^*CV*, where *V* is a column vector of length *K* with its *j*th element defined as $\frac {O_{j}-E_{j}}{\sqrt {E_{j}}}$, and *C*=*I*+*B*(*J*−*B*^*T*^*B*)^−1^*B*^*T*^ is a positive definite matrix in place of the identity matrix in the standard Pearson statistic. This leads to a $\chi ^{2}_{(K-1)}$ statistic. This has shown good power in simulations, and may be a good way to improve the power of our test in borderline cases.

The other issue that influences the degrees of freedom is the estimation of a maximum likelihood (ML) tree topology. This can be thought of as a discrete parameter space (so the combination of tree topology and other parameters is a mixed parameter space). These have been the topic of some research, beginning with Hammersley [18]. Choirat and Seri [6] give an account of the research done on this topic. From an asymptotic point of view, if the null hypothesis is correct, then by consistency, we know that with enough data, we are virtually certain to choose the correct tree, so the issue will not affect our degrees of freedom. However, in practice, consistency requires far more data to ensure the correct tree than the asymptotics for continuous parameters. Thus for our Chi-squared statistic, we should not just ignore the effect of tree selection. For most cases the probabilities of wrong trees converge to zero exponentially, but calculating the constant terms to apply these asymptotics is very difficult. Most of the literature focuses on very general upper and lower bounds, which are difficult to calculate, and of little use in our case. Given the high power we have achieved by using a conservative *K*−1 degrees of freedom test, it is hard to justify the use of a much more complicated test with increased computational complexity for the gainning of slight power in this case. We therefore avoid this problem here, by not removing any degrees of freedom for the tree search problem.

## Results

We will examine the effectiveness of the proposed test procedure through simulations and real data analyses.

### Simulation for equal frequency binning on a 4-taxon tree

The most obvious reason that site patterns in the same bin would be biased in the same direction is if the model mis-specifies the frequencies of the four nucleotides. Other biasses in estimating substitution rates should have less effect. We therefore consider the model SYM [42], which has the same exchangeability matrix as GTR, but is constrained by the assumption that the nucleotide frequencies are all 0.25. Our binning procedure should have good power to reject this SYM model, but might be less powerful when the data are generated under this SYM model. We also use a SYM+*D* model, which is this SYM model, but where the rates of evolution for each site follow a discretised gamma distribution (for illustrative purposes we simply generate 50% of the sites with one rate and the other 50% of the sites with a different rate).

**Fig. 1:**
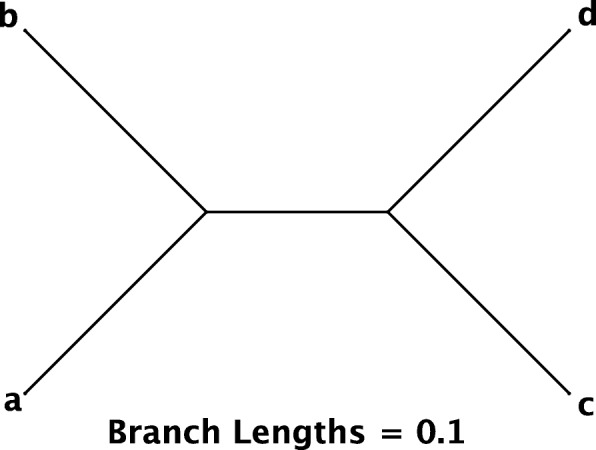
The 4-taxon tree used for simulation

200 data sets were simulated with sequence length 500 under each of the GTR, SYM and SYM+*D* models. The parameters for the GTR model were set to equal to the estimates from the *ψη*-Globin Pseudogenes data [41] : *π*_*T*_=0.308,*π*_*C*_=0.185,*π*_*A*_=0.308,*π*_*G*_=0.199; *r*_1_=0.987,*r*_2_=0.11,*r*_3_=0.218,*r*_4_=0.243,*r*_5_=0.395,*r*_6_=1. The exchangeabilities for SYM were the same as that of GTR but equal frequencies *π*_*T*_=*π*_*C*_=*π*_*A*_=*π*_*G*_=0.25 are assumed. The ratio of the branch lengths for generating each half of a single sequence for the SYM+*D* was 1:10. *INDELible1.03* was used for simulation. The tree used (shown in Fig 1) is an easy tree to estimate, so ML always found the correct tree. The simulation-analysis scenarios in this section are SYM-SYM, GTR-SYM, SYM-JC69, GTR-JC69, and SYM+*D*-SYM. (The first model in each pair is the model used to simulate the data, while the second model was used to analyse the data, and tested for adequacy).

Size is the probability that a test will indicate an effect when there is no such effect in the data. Power is the probability that a test will indicate an effect when there truly is such an effect in the data. Table 2 lists the results of the goodness-of-fit test with significance level *α*=5*%* for each scenario. In the SYM-SYM case, the goodness-of-fit test has a 5.5% rejection rate, so the size of the test is satisfactory. For the other cases with mis-specification of the models (GTR-SYM, SYM-JC69, GTR-JC69), the rejection rates are all approximately 100%. Hence, the power is also satisfactory. For the case SYM+*D*-SYM, the rejection rate is 31%, so the power of the test under this case is not as high as the other cases.

**Table 2:** The rejection rates out of 200 data sets for the goodness-of-fit test (Shortened as GoF in the table) based on equal frequency binning and the LRT for a 4-taxon tree, sequence length=500, *α*=5*%*

Rejection rate comparisons				
True model	*H*_0_	GoF test	LRT	*H*_*a*_ in LRT
SYM	SYM	5.5%	4.5%	GTR
GTR	SYM	98%	100%	GTR
SYM	JC69	100%	100%	GTR
GTR	JC69	100%	100%	GTR
SYM+*D*	SYM	31%	30.5%	SYM+*D*

The likelihood ratio test (LRT) can only be used for comparisons of two nested models; The LRT against the true model is known to be the most powerful test. As a benchmark, we compare the power of our test to the LRT. The null models, SYM and JC69, are both nested within the GTR model, thus the GTR model is used as the alternative model for most cases in the LRT test, except in the case where the true model is SYM+*D*, where we use the true model for the alternative. The size and power of the LRT are also included in Table 2. The results show that the size and power of our test is comparable to that of the LRT in all cases. Even in the case of SYM+*D*-SYM, the power of our test is as good as can be expected.

In this simulation study, the scenarios contain different degrees of model mis-specification. The size and power of the goodness-of-fit test are satisfactory for all cases in the simulation studies since they are similar to the LRT. Thus, this test seems to be a good tool for testing the adequacy of the model in the 4-taxon case.

### Size and Power of the test based on simulation for larger trees

**Fig. 2:**
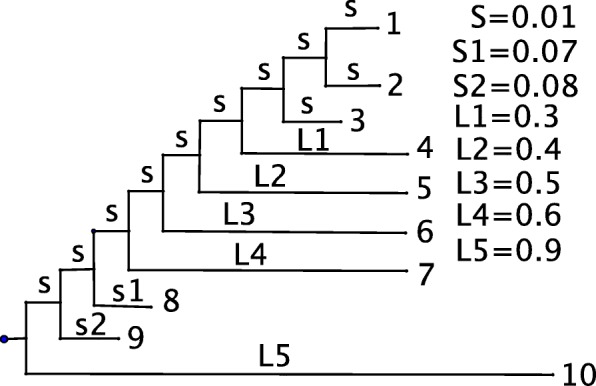
the 10-taxon tree topology used for simulation

A 10-taxon tree topology is used for generating DNA sequences. Figure 2 shows the tree topology with specified branch lengths. The design of the tree was made to give a relatively harder estimation problem. For example when we simulate 1000 data sets under the GTR model with sequence length 500, the estimated ML tree topologies only recover the true tree topology a small number of times under any model specifications (Table 3).

**Table 3:** The no. of ML tree topologies for 1000 data sets simulated under GTR model with sequence length 500, classified according to their Robinson-Foulds distances to the true tree topology

RF	0	2	4	6	8	10	12	14
JC	3	8	61	200	387	322	18	1
F81	1	17	83	238	362	277	20	2
HKY	58	135	230	279	189	97	12	0
GTR	86	195	257	230	150	68	13	1

We employ models GTR, F81, and GTR+ *Γ* to simulate data. Based on each generating model, using *INDELible1.03* we simulate 200 data sets for sequence lengths 500 and 200 respectively. The following model pairs are used to find the size and power of the test: (1) GTR-GTR (No model mis-specification — this will provide the size of the test), (2) GTR-JC69 (3) GTR-F81, (4) F81-JC69, and (5) GTR+ *Γ*-JC+ *Γ*.

**Fig. 3:**
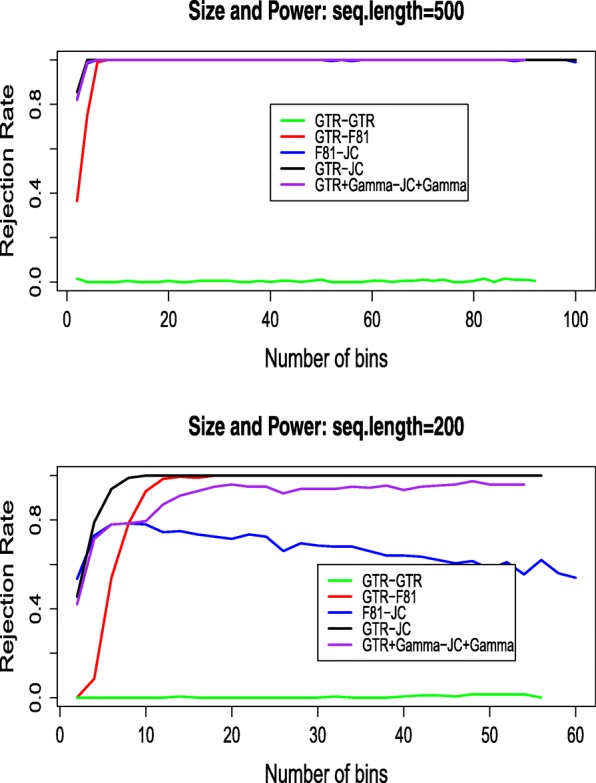
Rejection rates for different model pairs for seq. length 500 (upper) and 200 (lower)

In order to observe the effects of *K*, the number of bins, on the size and power of the tests, we analyze the simulated data for a range of different *K* values. For each *K* value, we calculate the percentage of times the tested model was rejected (at the 5% significance level) among 200 simulated data sets. The results are shown in Fig. 3.

From the results, we see that the size of the test is controlled very well, which is not surprising since the degree-of-freedom was chosen to make the test more conservative (see methods). The power of the test is related to the sequence length of the data and the model pairs. Generally when the sequence length is larger, the power of the test is higher. The power is lower for sequence length 200 for model pair F81-JC69. The choice of *K* is not critical for the performance of this test. For most of the model pairs, the power is high and stable (100%) for a wide range of *K* values when the sequence length is 500. When *K* is too small, the sites having upward or downward biases in their estimated probabilities will be naturally binned together, thus the test will lose power. A reasonable choice is to make *K* relatively larger but not so large that there are many bins with fewer than 5 expected sites.

**Fig. 4:**
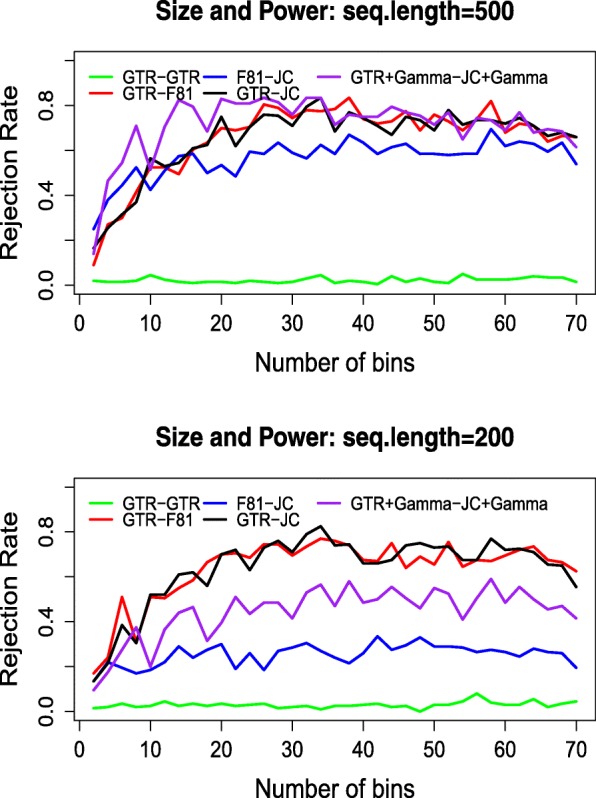
Rejection rates among 200 data sets for different model pairs under a random binning procedure, seq. length 500 (upper) and seq. length 200 (lower)

In order to illustrate the effect of the binning procedure on power, we compare the above results to a random binning procedure (see the Appendix A for the procedure of random binning). The size and power of the test under the random binning procedure is shown in Fig. 4. In comparison to Fig. 3, the results from random binning are much less stable, and the power of the test is much lower.

Table 3 also indirectly shows that by rejecting the inadequate model, we tend to get better tree topology estimation. Next we will show the difference in tree topology estimation by using an adequate or an inadequate model in a more direct simulation.

### Effects of the model adequacy test on tree topology estimation

**Fig. 5:**
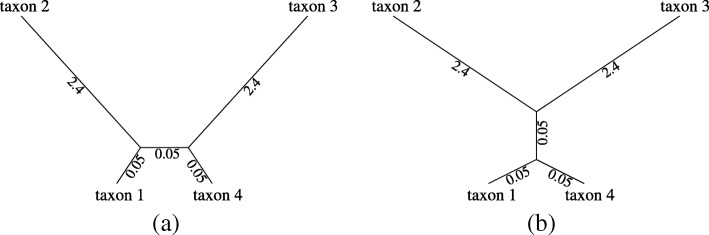
Trees used for assessing effect of adequacy on phylogeny estimation. **a** LBA tree. **b** “Anti-Felsenstein” tree

In this section, we use our model adequacy test to shed some further light on the effect of model mis-specification on estimation of the tree topology. We simulate data sets under a 4-taxon tree (Fig. 5a) which is a typical long branch attraction (LBA) problem. We simulate 2000 data sets for each sequence length 900 and 1800 nucleotides. Becuase we expect paramaters to vary among different real datasets we simulate the data sets with different parameters. For a simple illustration of this, we use two different sets of parameter values. In reality, we would expect parameter values to vary continuously, but this is more difficult to simulate and does not make the point of this simulation any clearer. In order to ensure that both the adequate and inadequate models are mis-specified, we simulate under a codon model, but analyse under F81+ *Γ*. We simulate using the codon frequencies estimated using different nucleotide frequencies in each codon position from the dataset “D2” of [40] which consists of 17 beta-globin sequences.

We use a model with equal exchangeabilities between nucleotides, but with double and triple changes of codons permitted. The rate of double changes is set to 0.06 times the rate of single changes, while the rate of triple changes is set to 0.03 times the rate of single changes. This model has previously been used in [11] for studying issues with codon methods. We use COLD [23] to simulate the datasets.

Although the codon model used for simulation is structurally different from the fully site-independent F81+ *Γ* model on the DNA level, our simulation, in the absence of selection pressure should generate a site pattern distribution fairly close to the distribution predicted by the F81+ *Γ* model. The second 1,000 datasets were simulated using the same parameters, except that in addition, we set the non-synomimous/synomimous ratio (*ω*) to 0.05. The effect of selection is to induce stronger dependence among the three positions of the codon, thereby generating a site pattern distribution very different from the pattern predicted by F81+ *Γ*. Note that because we simulate under a codon model, the branch lengths are the expected number of codon substitutions, which would be about three times the expected number of DNA substitutions.

**Fig. 6:**
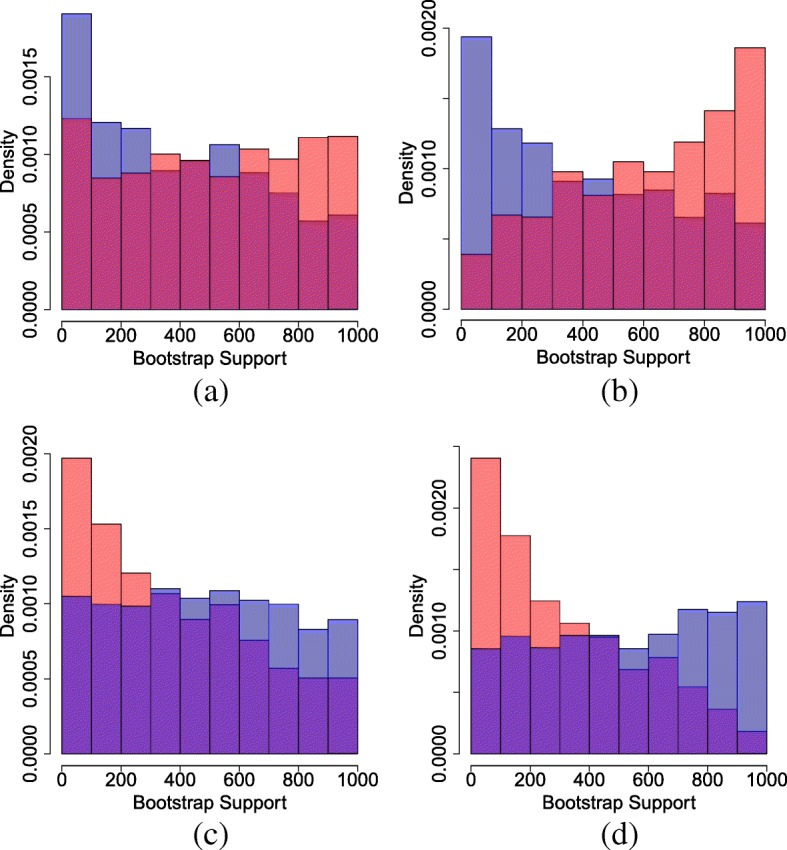
Comparison of boostrap support for the true and LBA trees under adequate models (red) and inadequate models (blue). **a** 900 nucleotides, true tree. **b** 1800 nucleotides, true tree. **c** 900 nucleotides, LBA tree. **d** 1800 nucleotides, LBA tree

We use F81+ *Γ* to analyze all 2000 data sets. We compute bootstrap support for each tree topology under the F81+ *Γ* model, separating the cases where F81+ *Γ* is rejected and where F81+ *Γ* is not rejected. (We use *K*=30 in our test to perform the model adequacy test.) The results are summarised in Fig. 6.

Of the 2000 data sets, F81+ *Γ* is rejected in 772 cases for sequence length 900, and in 1285 cases for sequence length 1800. We see that not only does the inadequate model incorrectly choose the LBA tree more often; it also often gives strong bootstrap support to the incorrect tree, meaning that we are falsely confident of this tree. By contrast, the adequate model not only prefers the true tree: even in cases where it selects the wrong tree, it gives lower bootstrap support to the LBA tree, showing us the level of doubt present in this estimate.

**Fig. 7:**
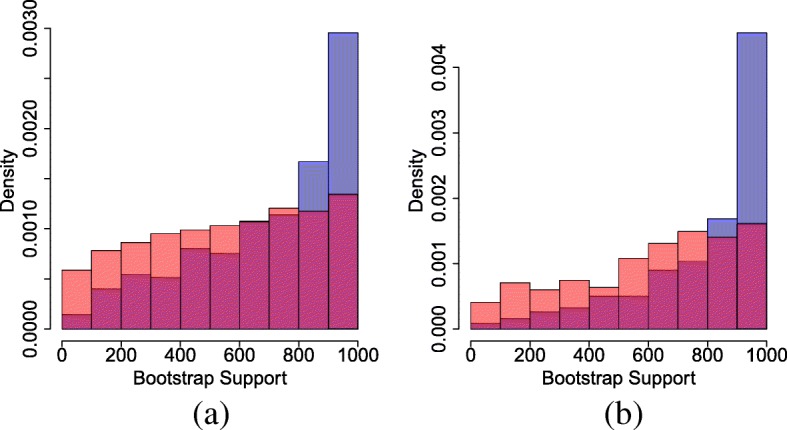
Comparison of boostrap support for the true tree in the “Anti-Felsenstein zone” under adequate models (red) and inadequate models (blue). **a** 900 nucleotides, true tree. **b** 1800 nucleotides, true tree

For completeness, we also compare a tree in the so-called “Anti-Felsenstein zone”, namely the case where the long branches are in a clade together (Fig. 5b). This is a case where many mis-specified methods are biased towards the truth. Figure 7 shows the results for this case. In this case, F81+ *Γ* is rejected in 623 simulations with sequence length 900 and in 1168 simulations with sequence length 1800. As expected the true tree is favoured in both adequate and inadequate cases. In the adequate cases, the bootstrap support shows the level of uncertainty about the tree, giving increasing support to the tree as the sequence length increases. The inadequate models give falsely confident support to the true tree because of the bias.

### Empirical data analysis

In this section, we use our goodness-of-fit test to assess the suitability of commonly used DNA models on a number of empirical data sets. The 23 empirical data sets used here are from the 25 empirical data in [30]. (We were unable to locate two data sets, 8 and 13, because Treebase was renumbered since [30] was published). Ripplinger and Sullivan [30] and Goldman [17] found that the GC test failed to reject the model JC+I+*Γ* for many empirical data, where the “I” represents the proportion of invariant sites, and the “ *Γ*” represents the among site rate variation. Here, we apply our proposed test on the same types of models as in the simulation analyses, but with invariant sites I and *Γ* rate variation added in the models. The null hypotheses for each of the data sets are:

**Table:** 

${H_{0}^{1}}$ JC+I+*Γ* is the true model;
${H_{0}^{2}}$ F81+I+*Γ* is the true model;
${H_{0}^{3}}$ HKY+I+*Γ* is the true model;
${H_{0}^{4}}$ GTR+I+*Γ* is the true model;

We have examined a range of *K*, starting at 2 until the rule-of-thumb for the Chi-square test are no longer satisfied. We have based our analysis on the largest *K* such that the rule-of-thumb is satisfied. In most cases, the conclusions are not sensitive to the choice of *K*. In cases where the conclusions are unclear, or appear contradictory (e.g. one model is not rejected, but a more complicated model is rejected), other values of *K* can provide a reference to help in our interpretation.

Under each hypothesis, the expected frequencies are estimated by simulating a DNA sequence with 100,000 sites based on a parametric bootstrap procedure.

The *p*-values for the goodness-of-fit tests are shown in Table 4. The *p*-values for the GC test for each data set from the supplementary material of [30] are recorded on the right side of Table 4 for comparison.

**Table 4:** *p*-values for various models and various data sets (from Ripplinger and Sullivan, 2010)

For each data set, the largest value of *K* was chosen, so that the rule-of-thumb for Pearson’s Chi-square test was not violated for all four tests. Models which could be rejected by our method are highlighted in yellow. Green shade is used to indicate uncertainty caused by conflicting results for different *K*-values and other models. [Treebase numbers differ from those listed in Ripplinger and Sullivan (2010) because Treebase renumbered its data sets since that paper was published. The listed sequence length is the no. of sites used in analysis after all gaps are removed.]

The results of the new test suggest that in most cases JC+I+*Γ* is not adequate. In many cases, even GTR+I+*Γ* is not adequate.

There is an interesting phenomenon in data sets 14, 15, 20, 24 and 25, where GTR+I+*Γ* can be rejected, but simpler models cannot. We can gain more insight into these cases by examining the results for different values of *K*. For data set 14, for a range of *K* values (*K*=6–16), we can reject GTR+I+*Γ* all at *p*=0. We can only reject HKY+I+*Γ* when *K*=12,13,15 and 17. The largest *K* value for which the rule-of-thumb for Pearson’s Chi-square test was not violated for testing HKY+I+*Γ* is *K*=17 and the corresponding *p* value is 0.0039. Given the strong evidence to reject GTR+I+*Γ* and slightly weak indication of rejection of HKY+I+*Γ*, we conclude that all models are inadequate for this data set.

For data set 15, taking *K*=8, we can reject both F81+I+*Γ* and HKY+I+*Γ* (*p*=0.009 and *p*=0.03 respectively). Taking *K*=7, we can reject F81+I+*Γ* (*p*=0.025) but not HKY+I+*Γ* (*p*=0.11). The largest *K* value for which the rule-of-thumb is satisfied for both F81+I+*Γ* and HKY+I+*Γ* models is 8. This suggests that these models should be rejected. This data set has 35 taxa and the sequence length after removing gaps is 333. With the relatively small *K* values for which the goodness-of-fit test can be performed, the power of the test tends to be lower. This conclusion should be checked when more data become available.

For data set 20, taking *K*=11, we can reject HKY+I+*Γ* (*p*=0.042), but HKY+I+*Γ* cannot be rejected at any other *K* values. GTR+I+*Γ* can be rejected for all *K* values between 6 and 14, all with very small *p*-values. This suggests that these models should all be rejected. From the result of this data, it seems the power of the test is slightly lower for HKY+I+*Γ* model than for the GTR+I+*Γ* model.

For data set 24, we cannot reject F81+I+*Γ* or HKY+I+*Γ* for almost all different *K* values, except when *K*=17, we can reject F81+I+*Γ* at *p*=7.6*e*−5. GTR+I+*Γ* can be rejected for all *K* values ranging from 11 to 16. It is not clear in this case if all these four models are inadequate or the rejection of GTR+I+*Γ* at these *K* values is only due to random errors.

For data set 25, for *K* values ranging from 6 to 30, both F81+I+*Γ* and HKY+I+*Γ* models are all rejected at the 5% level. Although the *p*-values for *K* between 31 to 46 for models F81+I+*Γ* and HKY+I+*Γ* are more variable, both models can be rejected in most of the cases. GTR+I+*Γ* can be rejected for all *K* values with quite small *p*-values. This suggests that all these four models should be rejected.

Among 23 data sets, there are 6 data sets with sequence length between 300 and 400 (data set 3,5,6,11,15,17) and no. of taxa ranging between 17 and 75. The largest *K* values for which the rule-of-thumb can be satisfied when sequence length is small tends to be low, and the number of different site patterns for such a high number of taxa is very large. This combination means it is almost impossible to bin the site patterns so that biases are in the same direction within each bin, thus the test power is naturally low. One of the conflicting result cases (data set 15) is among this group. The tests for the other five cases (data sets 3,5,6,11,17) all either fail to reject any model, or reject only JC+I+*Γ*. In these cases, because the data sets are relatively small, there may not be sufficient data to perform inference or model selection, so the fact that the models are adequate does not mean that the conclusions from them are good, but rather that there may not be enough data to draw reliable conclusions at all (even with a better model). Standard inference techniques (e.g. bootstrapping and topology tests) will indicate in this case that there is a great deal of uncertainty about the conclusion. However an adequate model indicates that one should not expect to reach better conclusions by choosing a more complicated model, even if it is closer to the true model.

In general, the adequacy test does not replace usual inference, but rather complements it — testing both possible sources of inaccuracy, i.e. bias and variance. Using an inadequate model typically causes large bias in the conclusion. Thus if a bootstrap gives confident support to a particular conclusion, but the model is inadequate, the conclusion is still unreliable. In this case the model adequacy test will give some indication of the additional uncertainty caused by using an incorrect model. When using an adequate model to perform the analysis, the inaccuracy of the conclusion can be inferred using the variance.

### Assessment of binning site with biases in the same direction

As mentioned earlier, any binning method will produce some capacity to test model adequacy, but it could have low power. The objective in devising the binning procedure is to bin together site patterns whose probability estimates will mostly be biased in the same direction — i.e. site patterns for which the probabilities will all be overestimated should be binned together, as should site patterns for which the probabilities will all be underestimated. The expected probabilities for each bin are calculated as the sum of the probabilities of all site patterns in the same bin under the null model. If all or most of the site patterns in a bin are biased in the same direction under the wrong null model, the difference between the observed frequencies and expected frequencies for the bin will naturally be large.

To test how effective our binning procedure has been at achieving this objective, we perform another simulation. For a general adequacy test, we hope to achieve this objective for a wide range of different simulation models. Therefore in addition to the parameters for the rate matrix we have used in the above simulation, we also get the estimated GTR model parameters from the 23 real data sets analyzed in the above “Empirical data analysis” section. For each set of GTR model parameters, we simulate 1000 data sets under the GTR model, each with sequence length 1000, using the 4-taxon tree from Fig. 1. We then analyze the simulated data sets under F81, and calculate the probability of each site pattern under the estimated F81 parameters. The bias in the estimated probability for each site pattern is calculated as the difference between the mean of the estimated site pattern probabilities under F81 over 1000 data sets and the site pattern probability under the generating model (GTR) and the true model parameters.

**Fig. 8:**
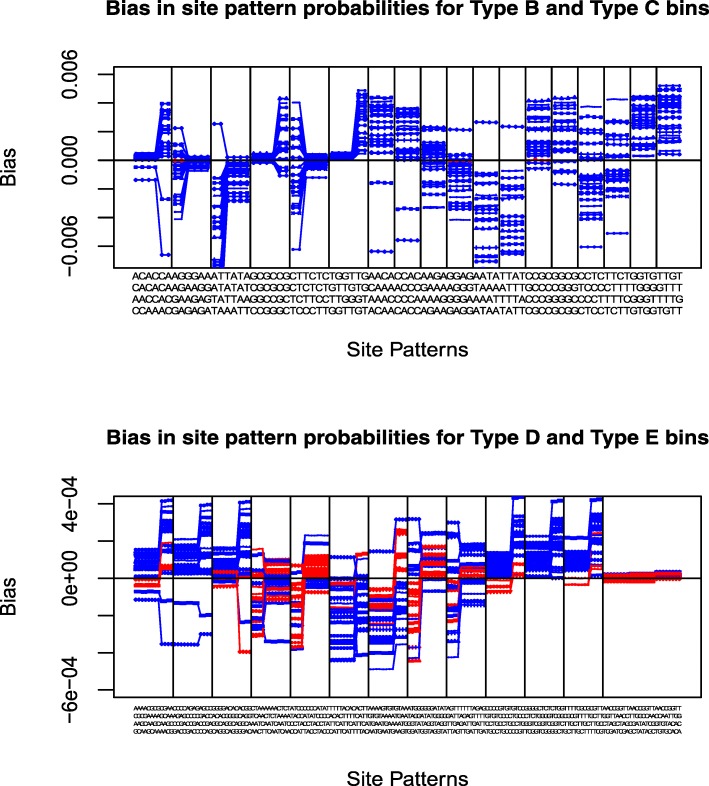
Bias in estimating expected probabilities of site patterns in each bin: Type B and Type C bins in the upper panel, Type D and Type E bins in the lower panel. Blue lines label the biases in the same direction for all site patterns in a bin, red lines label the biases are not in the same direction

We look at the bias in the estimated probabilities for all the site patterns, and we compare these biases for the site patterns in each bin. For Type A bins (See Table 1), because each bin only contains one site pattern, it is not necessary to look if the site patterns are biased in the same direction in each bin. We present the results for Type B and Type C bins in the upper panel and Type D and Type E bins in the lower panel of Fig. 8. Within each bin of Fig. 8, there are 24 connected lines, each of which represents the bias in the site pattern probabilities for all the site patterns within this bin for one set of GTR model parameters. Blue lines mean that all biases are in the same direction, red lines mean some site patterns biased up and some site patterns biased down. Almost all bins for Type B and Type C contain purely blue lines for all 24 sets of model parameters. For Type D and Type E bins, there are some bins with mixed biases for a few sets of models. Note that it is not reasonable to expect to bin sites in such a way that all site patterns are always biassed in the same direction in all bins for any true model settings. The bins we have chosen are performing fairly well overall. In many of the cases of mixed bins, the biasses are smaller than the other bins, and in many cases, the bias is particularly small on either the positive or negative side. This means that the overall bias for the bin can still be fairly large, which gives the Chi-square test good power. This explains why we perform better than a random binning. In summary, we see that our binning procedure has done a good job of binning together site patterns which show a similar direction of bias. We therefore expect our binning procedure to perform well for testing the model adequacy.

It is worth noting that the model mis-specification in this simulation is the use of F81 instead of GTR, so there is no model mis-specification for the nucleotide frequencies. Nevertheless, our binning, based on counting the frequency of each nucleotide in the site pattern, still groups the site patterns well. The explanation for this is that the mis-specification in this case causes the rate of various point mutations to be misestimated. The frequency of each nucleotide often provides a good indication of which nucleotide mutations have taken place. For example, if a site pattern involves only the nucleotides A and C, then we know that the probability of this site pattern is related to the rate of exchangeability between A and C, so if, for example, the mis-specification causes this to be underestimated, the probability of the site pattern will also often be underestimated.

## Discussion

Our goodness-of-fit test has shown very good power and size, both for the small tree simulation where exact frequency binning of site patterns was used; and for the larger tree simulation where *K*-means clustering was used. Our method also showed good power in the real data examples. The power remains high for a large range of values of *K*. We have shown that our binning procedure helps to improve the power of the test compared to binning the sites at random, because sites with similar biases are binned together. There is still potential to find better binning schemes that might be able to provide even better power, however the simplicity of the procedure may be compromised which may influence the applicability of the testing procedure. The design of the binning procedure is to make the test particularly sensitive to mis-specification for the nucleotide frequencies. Nevertheless, the simulation results show that even when nucleotide frequencies are correctly specified, the test still has good power to reject models with incorrectly specified exchangeability matrices.

It is well known that incorrect models can lead to wrong phylogenetic inferences (e.g. [35, 37]). Furthermore, models that are “closer to the truth” will usually produce better inferences. We demonstrated how model adequacy testing can help in this situation by providing better assessments of the reliability of the estimated tree. When an adequate model is used, phylogeny estimation tends to select the best tree topology according to the data, with inference methods such as bootstrap support giving a fair assessment of the uncertainty in the inferred tree topology. In cases where the model fails the adequacy test, the estimated phylogeny may be biased towards a particular topology, and the bootstrap support can strongly support this tree whether or not it is the correct tree. In general, the adequacy test does not replace usual inference, but rather complements it — we need to test for both possible sources of inaccuracy, using the adequacy test to check for bias and the usual inferrence methods (e.g. bootstrap support) to check the variance. Thus if a bootstrap gives confident support to a particular conclusion, but the model is inadequate, the conclusion is still unreliable. In this case the model adequacy test will give some indication of the additional uncertainty caused by using an incorrect model. When using an adequate model to perform the analysis, the inaccuracy of the conclusion can be inferred using standard inferrence.

Some recent research on Posterior Predictive Simulations (e.g. [3]) has speculated that by selecting a statistic for PPS that is closely related to the quality of tree estimation, it might be possible to devise a test which is particularly sensitive to cases that are likely to cause mis-estimation of the tree. While this would be desirable, since for many analyses the inferred tree is the main interest, there is little evidence that a powerful generalised test for the quality of tree estimation can be developed. Because tests that can specifically identify mis-specifications that cause phylogenetic error remain elusive, general tests of model adequacy remain an important area of research in phylogeny.

## Conclusion

We have developed a procedure to bin site patterns in order to apply Pearson’s goodness-of-fit test for DNA substitution models in phylogenetic analysis. The null hypothesis is that the substitution process follows a given model. The binning procedure is based on the frequencies of each nucleotide in a site, and the use of *K*-means to cluster similar site patterns. Based on our simulation studies and real data analysis, this test has shown good power to reject wrong models across a wide range of scenarios. Further work could still be done to gain a more complete understanding of the scenarios where we expect this method to work best.

We have explained why our binning procedure is superior to binning the site patterns at random, and provided some insight into what makes a good binning procedure. Further study on this topic could lead to improved methods for developing goodness-of-fit tests for phylogenetic models.

We discuss the topic of degrees of freedom. We have taken a conservative view that it is appropriate to use *K*−1 degrees of freedom to be certain of controlling Type I errors. However, obtaining a better distribution of the test statistic by parametric bootstrapping, or using the approach from [29] to get an improved test statistic might lead to better results. Neither of these approaches deals with the effect of estimating the tree topology. This is a difficult statistical problem, and requires substantial work in statistical theory.

More generally, the effect of tree estimation on the results warrants further investigation in future studies of model adequacy. If a model is severely mis-specified, it might estimate a very poor tree, and this could adversely influence the results.

From the real data analysis, we see that in many of the data sets, even GTR+I+*Γ* is not adequate. This indicates that the inference results of any nucleotide-based analysis on these data sets should not be considered entirely reliable. Nucleotide-based analyses on the data sets where we could not reject most nucleotide models are expected to lead to reliable inference results.

## Appendix A: The random binning procedure

The procedure for random binning to produce *K* bins is as follows:

- For each taxon, assign a random value 0 to *K*−1 to each nucleotide (A, C, G, T)
- For each site, add up the numbers assigned to the nucleotides for that site
- Take the remainder upon division by *K*.

For example, for *K*=7, each of the following four taxa was assigned a rule generated by random draws from 0 to 6. Then for site one, the sum of the scores is 2+3+3+0=8 thus this site is assigned to bin 1. Similarly, site 2 will be assigned to bin 1 too, and sites 3 and 4 will be assigned to bins 2 and 6 respectively.

**Table:** 

Taxon	A	C	G	T	Sequence
Taxon 1	2	4	3	3	ACTG…
Taxon 2	3	4	6	0	AATC…
Taxon 3	1	6	3	1	GCTC…
Taxon 4	5	2	0	5	GCAG…

## Appendix B: Proof of Theorem 1

### **Theorem**

If the data include *n* sites, we divide the site patterns into *K* bins which satisfy the rule of thumb that the expected number of sites in each bin is at least 5, and we simulate *M* points, then the mean squared error in our estimated chi-squared statistic due to this simulation is at most

$$\frac{16Kn}{25M}$$

### *Proof*

Suppose the correct probability for the *i*th bin is *p*_*i*_. Let *Q*_*i*_ be the estimated proportion of sites in the bin. The number of simulated sites in the bin follows a binomial distribution with parameters *M* and *p*_*i*_, so *Q*_*i*_ has mean *p*_*i*_ and variance $\frac {p_{i}(1-p_{i})}{M}$. The part of the Chi-square statistic from the *i*th bin should therefore be $\frac {(O_{i}-{np}_{i})^{2}}{{np}_{i}}$, but our estimate is $\frac {(O_{i}-{nQ}_{i})^{2}}{{nQ}_{i}}$. The error in our chi-square statistic from the *i*th bin is therefore $\frac {(O_{i}-{nQ}_{i})^{2}}{{nQ}_{i}}-\frac {(O_{i}-{np}_{i})^{2}}{{np}_{i}}=\frac {({O_{i}^{2}}-n^{2}p_{i}Q_{i})(p_{i}-Q_{i})}{{np}_{i}Q_{i}}$. We will let *Q*_*i*_=*p*_*i*_+*E*_*i*_, where *E*_*i*_ has mean 0 and variance $\frac {p_{i}(1-p_{i})}{M}$. For large *M*, we can discount terms in *E*_*i*_^2^, so the error in our chi-square statistic is $\frac {({O_{i}^{2}}-n^{2}{p_{i}}^{2})E_{i}}{n{p_{i}}^{2}}$. The mean squared error is therefore

$${} \left(\frac{\left({O_{i}^{2}}-n^{2}{p_{i}}^{2}\right)}{n{p_{i}}^{2}}\right)^{2}{\mathbb E}({E_{i}}^{2})=\left(\frac{\left({O_{i}^{2}}-n^{2}{p_{i}}^{2}\right)}{n{p_{i}}^{2}}\right)^{2}\frac{p_{i}(1-p_{i})}{M}$$

If we assume the errors for each class are independent (which is clearly not totally true since the errors sum to 0, but for a reasonable number of classes, should be acceptable) then the total MSE of our estimated chi-squared statistic is

$$\sum_{i=1}^{K}\left(\frac{({O_{i}^{2}}-n^{2}{p_{i}}^{2})}{n{p_{i}}^{2}}\right)^{2}\frac{p_{i}(1-p_{i})}{M}$$

Since under the null hypothesis, *O*_*i*_ follows a binomial distribution with parameters *n* and *p*_*i*_, we have that $ {\mathbb {E}}\left ({O_{i}}^{2}-n^{2}{p_{i}}^{2}\right)={np}_{i}(1-p_{i})$ and

$${} {\mathbb{E}}\left(\left({O_{i}}^{2}-n^{2}{p_{i}}^{2}\right)^{2}\right)={np}_{i}(1-p_{i})\left(3(n-2)p_{i}(1-p_{i})+1\right)$$

The expected total MSE is therefore

$${\begin{aligned} &\sum_{i=1}^{K}\left(\frac{{np}_{i}(1-p_{i})\left(3(n-2)p_{i}(1-p_{i})+1\right)}{n^{2}{p_{i}}^{4}}\right)\frac{p_{i}(1-p_{i})}{M}\\ =&\sum_{i=1}^{K}\left(\frac{3(n-2){p_{i}}(1-p_{i})^{3}+(1-p_{i})^{2}}{Mn{p_{i}}^{2}}\right)\\ \,=\,&\frac{1}{M}\left(\frac{3(n-2)}{n}\sum_{i=1}^{K}\left(\frac{1}{p_{i}}-3\,+\,3p_{i}-{p_{i}}^{2}\right)\,+\,\frac{1}{n}\sum_{i=1}^{K}\frac{(1-p_{i})^{2}}{{p_{i}}^{2}}\right) \end{aligned}} $$

Assuming the rules of thumb for the chi-square test are satisfied, we must have ${np}_{i}\geqslant 5$, so that $\sum _{i=1}^{K}\frac {1}{p_{i}}\leqslant \frac {Kn}{5}$ and $\sum _{i=1}^{K}\frac {1}{{p_{i}}^{2}}\leqslant \frac {Kn^{2}}{25}$. The MSE in our chi-square statistic due to using simulation is therefore bounded by

$${\begin{aligned} &\frac{1}{M}\left(\frac{3(n-2)}{n}\left(\frac{Kn}{5}-3K+3-\frac{1}{K}\right)+\frac{K}{n}\left(\frac{n}{5}-1\right)^{2}\right)\\ =&\frac{1}{25M}\left(15K(n-2)\,-\,225K\frac{n-2}{n}\,+\,225\frac{n-2}{n}-\frac{75(n-2)}{Kn}\,+\,Kn-10K+\frac{25K}{n}\right)\\ =&\frac{1}{25M}\left(16Kn-265K+475\frac{K}{n}+225-\frac{450}{n}-\frac{75}{K}+\frac{150}{Kn}\right)\\ \leqslant&\frac{16Kn}{25M} \end{aligned}} $$

where the last inequality assumes $K\geqslant 2$ and $n\geqslant 2$. □

## Abbreviations

AIC_c_: Corrected Akaike information criterion
BIC: Bayes information criterion
COLD: Codon optimal likelihood discoverer
DT: Decision-theoretic
GC: Goldman-cox
GoF: Goodness of fit
hLRT: Hierarchical likelihood ratio tests
LBA: Long branch attraction
LRT: Likelihood ratio test
ML: Maximum likelihood
MLE: Maximum likelihood estimator
MSE: Mean square error
PPS: Posterior predictive simulations
